# A Critical Examination of the Clinical Diagnosis of Functional Tic‐like Behaviors

**DOI:** 10.1002/mdc3.14150

**Published:** 2024-06-28

**Authors:** Kaja Andersen, Andrea Eugenio Cavanna, Natalia Szejko, Kirsten R. Müller‐Vahl, Tammy Hedderly, Liselotte Skov, Nanette Mol Debes

**Affiliations:** ^1^ Department of Pediatrics Copenhagen University Hospital‐Herlev and Gentofte Herlev Denmark; ^2^ Department of Clinical Medicine University of Copenhagen Copenhagen Denmark; ^3^ Department of Neuropsychiatry BSMHFT and University of Birmingham Birmingham UK; ^4^ School of Life and Health Sciences, Aston Brain Centre Aston University Birmingham UK; ^5^ School of Medicine and Surgery University of Milano‐Bicocca Milan Italy; ^6^ Department of Psychiatry, Social Psychiatry and Psychotherapy Hannover Medical School Hannover Germany; ^7^ Department of Clinical Neurosciences University of Calgary Calgary Alberta Canada; ^8^ Department of Bioethics Medical University of Warsaw Warsaw Poland; ^9^ Guys and St Thomas Hospital and KCL Faculty of Life Sciences and Medicine Evelina London Children's Hospital London UK

**Keywords:** functional tic‐like behaviors, functional neurological disorders, Tourette's syndrome, tic disorders

## Abstract

**Background:**

Since the COVID‐19 pandemic, movement disorder clinics have seen an increase in patients with an unusual type of tic‐like symptoms: young adults with abrupt onset complex behaviors. It was quickly suspected that these patients suffered from functional neurological symptoms, later named Functional Tic‐Like Behaviors (FTLB). Subsequent research on the differential diagnosis between FTLB and tics has been substantial and led to the development of diagnostic checklists.

**Objectives:**

We conducted a theoretical reappraisal of the FTLB literature to clarify the validity of the concept and its diagnostic implications.

**Methods:**

This paper addresses several key aspects of the current FTLB literature: circular reasoning, the complications of the FTLB phenomenology and demographics, the impact of FTLB on tic literature at large, and issues with alignment of the FTLB concept with the diagnostic criteria for functional disorders.

**Results:**

The clinical approach to FTLB might involve circular reasoning due to a lack of clinical benchmarks. The FTLB phenomenology and demographics may need more work to ensure a lack of bias and a proper description of this patient group including a clear distinction from tics. The impact of the FTLB discussion on the wider literature needs consideration. The validation of positive signs may help with both these endeavors and pave way to the inclusion of FTLB within psychiatric classification systems. Furthermore, the coexistence of FTLB and tics within the same patient needs to be addressed.

**Conclusion:**

More research may be needed to fully establish the diagnosis of FTLB and differentiate it from tics.

During the COVID‐19 pandemic, movement disorder clinics worldwide saw an increase in the number of teenagers and young adults referred with an abrupt development of severe and complex movements and vocalizations.^1^ These patients attracted considerable attention due to the notable differences between their presentation and the one seen in tic disorders (TD) such as Tourette's Syndrome (TS), where patients typically have a gradual childhood onset with predominantly simple movements and vocalizations.^2^, ^3^ Based on previous studies, it was asserted that the movements and vocalizations of a number of these adolescents were not symptoms of a TD but rather of functional tic‐like behaviors (FTLB), a presentation of a functional neurological disorder (FND).^4^ Much of the subsequent research within this area has focused on delineating the differences between patients with FTLB and patients with TD. The resulting findings have led to detailed characterizations of both groups that are summarized in Table 1.

**TABLE 1:** Characteristics of patients with FTLB and TD.^1^, ^2^, ^4^, ^5^, ^6^, ^7^, ^8^, ^9^, ^10^, ^11^, ^12^, ^13^

	Patients with FTLB	Patients with TD
Sex	Predominantly female	Predominantly male
Age	Older than 12 years at symptom onset	Younger than 12 years at symptom onset
Family history of tics	Not commonly	Commonly
Comorbidities	Typically anxiety and depression	Typically ADHD and OCD
Symptom onset	Rapid progression	Gradual progression
Phenomenology	Predominantly complex movements and vocalizations with lack of rostrocaudal distribution	Predominantly simple tics with rostrocaudal distribution
Presence of socially inappropriate and harmful behaviors	Frequent presence of harmful behaviors including socially inappropriate words and gestures (coprophenomena), self‐harming behavior, or behavior that harms others	Rare occurrence of harmful tic behaviors

Abbreviations: ADHD, attention deficit hyperactivity disorder; FTLB, functional tic‐like behavior; TD, tic disorder; OCD, obsessive compulsive behaviors.

Recently, two diagnostic checklists for FTLB have been proposed based on these characterizations. Pringsheim and colleagues suggest three major criteria that are mandatory for a clinical definite diagnosis of FTLB: older age than 12 at symptom onset, abrupt symptom onset, and presence of at least four out of nine different listed tic‐like behaviors, consisting of complex and harmful behaviors, along with atypical timing and distribution of the symptoms.^14^ Additionally, their checklist also includes two minor criteria, which are not mandatory but do increase the likelihood that the patient suffers from FTLB: presence of anxiety and depression as comorbidities and presence of other functional symptoms. A clinical probable diagnosis of FTLB can be reached through the combination of one of these minor criteria and two of the major ones. Trau and colleagues have also developed a checklist for the diagnosis of FTLB, resembling the one by Pringsheim and colleagues,^14^ except for their inclusion of female sex as a criterion.^10^

This research effort into the differential diagnosis between FTLB and TD and the development of diagnostic checklists for FTLB stems from an understandable desire to avoid misdiagnosis. FTLB's status as a clinical phenotype of FND implies that its etiology is fundamentally different from TD,^15^, ^16^ and this difference in origin translates to a difference in how the patients should be treated. For example, the psychoeducation provided to patients needs to have different content, and patients with FTLB should not be prescribed the pharmacotherapy used for TD, as this would be ineffective and potentially harmful.^1^, ^6^

Due to these important differences in treatment, clinicians will benefit from diagnostic checklists that can assist in the differential diagnosis between TD and FTLB with a high level of precision. As such, it is important that the current understanding of FTLB that feeds into the diagnostic checklists is critically examined, so that a higher level of accuracy can be ensured.

## Methods

This article aims to provide an examination of the current understanding of FTLB by outlining the current issues within the FTLB literature and critically appraising unavoidable problems of circular reasoning and flawed characterizations of both TD and FTLB. Finally, the relationship between FTLB and the wider FND literature will be explored.

## Results

### Circular Reasoning

The first problem with the FTLB literature is incomplete reporting of the diagnostic process. Most studies on FTLB patients do not describe which characteristics or other patient factors were used by clinicians to make the diagnosis.^5^, ^6^, ^7^, ^8^, ^9^, ^11^, ^12^, ^13^ This oversight has several consequences for the interpretation of these studies’ results. For validation of the FTLB characterization, a key issue is that the lack of reporting makes it impossible to tell whether the characteristics explored in the studies’ analyses are the same used to diagnose the patients.^1^ If the same characteristics used to diagnose patients are the ones being tested statistically to differentiate between TD and FTLB, this could threaten the validity of the results, as they would not be pathognomonic features of FTLB but instead stem from the diagnostic process. The only scientific article where the presence of this type of circular reasoning can be transparently assessed is the study by Trau and colleagues published in 2022, as this research group does report which characteristics the initial FTLB diagnosis was based on.^10^ In this paper, presence of “tic attacks” results in a diagnosis of FTLB, but “tic attacks” are also included as a part of the analysis of differences between patients with FTLB and TD. Based on the result of their analysis, Trau and colleagues conclude that there is an increased prevalence of “tic attacks” in patients with FTLB, when it seems likely that this result was due to their initial categorization of the patients. As the remaining FTLB literature suffers from a lack of reporting of the initial diagnostic criteria and procedure, it is difficult to determine to what extent, if any, similar circular reasoning is responsible for other findings surrounding the characterization of patients with FTLB and TD.

### The Validity of the Patient Characterizations

#### The Complex Nature of Tic Phenomenology

As outlined in the introduction, the phenomenology of FTLB includes complex tic‐like movements and/or vocalizations including coprophenomena, “tic attacks,” and potentially harmful behaviors such as self‐injurious behavior and destructive behavior directed towards objects or other people.^1^, ^2^, ^4^, ^5^, ^6^, ^7^, ^8^, ^9^, ^10^, ^11^, ^12^, ^13^ Patients must display several of these behaviors to be classified as a clinically definite case of FTLB in both diagnostic checklists, as no behavior is considered pathognomic in isolation. Instead, a constellation of symptoms is used as a major differential factor to distinguish FTLB from TD.^10^, ^14^ However, this complex phenomenology is also seen in some patients with TD, whereas some patients with FTLB do not exhibit it, making the claim that it is a defining trait of FTLB more complicated.

While most publications on FTLB do not provide the count of each patient's complex tic‐like symptoms, the available data raises the possibility that a significant portion of the FTLB population have no or very few complex symptoms, in contrast to the phenomenology described in the literature. Fifteen percent of the 294 patients with FTLB included in a retrospective international registry of the patient group had no complex movements, while 19% had no complex vocalizations.^7^ In another independent cohort (N = 53), 18% of the patients with FTLB had no complex movements, 44% had no complex vocalizations, and 15% had no complex behaviors at all.^5^

Furthermore, most articles exploring the prevalence of coprophenomena, “tic attacks,” and harmful behaviors in patients with FTLB find that the majority of these behaviors are seen in less than half of the population and that none of the behaviors are seen in all of the patients.^7^, ^9^, ^10^, ^11^ Anecdotally, 34% of the patients with FTLB in the Danish cohort exhibit none of the recorded behaviors (coprophenomena, unrestrained speech, “tic attacks,” and self‐injurious behavior).

Based on these numbers, there seems to exist a subset of the FTLB population whose symptoms do not fit with the established FTLB phenomenology, suggesting that while FTLB checklists provide useful indicators, using them as strict guidelines for all patients may be detrimental.

The existence of patients with TD who exhibit symptoms resembling FTLB phenomenology has been documented within the TD literature for many years. Studies which divide patients with TD into different groups based on the characteristics of their tics have consistently found a subgroup of patients with severe complex tics and high prevalence of harmful behaviors.^17^, ^18^ Furthermore, a similar subgroup of patients is described in studies on “malignant tic disorder” or “self‐injurious tics,” which delineates a clinical phenomenology with severe and complex tics (including dystonic tics), self‐injurious behaviors, rage attacks, and coprophenomena.^19^, ^20^, ^21^, ^22^ Thus, there seems to be an established subset of patients with TD who have a clinical phenomenology similar to the one described for FTLB. As this TD population would thus meet at least the checklists’ tic criterion, there is a risk that patients in this subgroup could be misdiagnosed with FTLB, particularly if the diagnostic checklists were used by physicians with less experience in tic disorders. This risk of misdiagnosis, when the checklists are in less experienced hands, increases even more if acute symptom onset and onset after age 12 are treated as salient criteria for FTLB,^10^, ^14^ despite the existence of a subset of patients with TD who share both characteristics.^23^ It should also be mentioned that the DSM/ICD criteria^24^, ^25^ used to diagnose TS and other TDs do not list onset before 12 years of age as a diagnostic criterion for tics attributed to neurodevelopmental origin and do not address acute‐versus‐gradual onset.

Apart from the atypical FTLB and TD patient groups discussed above, attempts to understand and characterize FTLB and TD phenomenology are also challenged by a third patient group: patients with both FTLB and TD. Previously, only a few case reports describing these patients existed,^26^, ^27^ but recently a bigger study including 71 patients was published.^28^ While the diagnostic criteria used in this study may be partly responsible for the reported patient phenomenology, it is notable that the phenomenology described in this study closely resembles classic FTLB, with complex behaviors, copropraxia and coprophenomena, self‐injurious behaviors, and tic attacks. Furthermore, the behaviors lacked rostrocaudal distribution, could be triggered by a change of context such as a new person entering the room, and were resistant to tic medication. Also, similarly to the classic FTLB patients, the TD + FTLB patients described their premonitory urge as a more global than the TD patients, and they had a higher rate of OCD^28^. Currently, the literature does not describe any attributes which may help to set TD + FTLB patients apart from pure FTLB which is problematic considering that the difference in visibility and severity of FTLB and TD could mean that some TD patients may only pursue help after their FTLB debut. It seems important that these patients are not confused with pure FTLB patients. Similarly, it is possible that some patients previously diagnosed with severe and/or treatment resistant TD, and described as such in the literature, may be suffering from both TD and FTLB, with their most eye‐catching symptoms stemming from the latter.

Considering the problems with the classic FTLB phenomenology's lack of applicability to all FTLB patients and the overlap between the symptoms of TD + FTLB, FTLB only, and severe TD, it is important that FTLB phenomenology is further discussed.

#### A Conservative Redefinition of the Phenomenological Boundaries between FTLB and TD

Another interesting point regarding the discussion of FTLB phenomenology is its impact on how tics and TD are generally conceptualized. Historically, it has been accepted that TDs can be expressed in a variety of ways, some more typical than others, and to various extents of complexity and severity.^23^, ^29^, ^30^, ^31^, ^32^ A pre‐pandemic article by Ganos and colleagues provides one of the clearest examples of this flexibility in the boundaries of tic definition and tic expression: “any biomechanically possible movement with muscle activation parameters within those accessible for normal voluntary movement may occur as a tic behavior.”^33^

The FTLB literature seems to challenge this inclusive definition and conceptualization of TDs. As a result, TD phenomenology is now focused on the milder and less complex phenomenology—those with predominantly simple tics and little‐to‐no harmful behaviors.^5^, ^6^, ^7^ More complex and atypical presentations, such as those with abrupt onset or non‐rostro‐caudal distributions, all of which have previously been recognized as TD variants, have been re‐conceptualized as expressions of FTLB.^10^, ^14^, ^19^, ^22^, ^23^, ^34^, ^35^ Furthermore, many of the complex behaviors which were previously thought of as possible symptoms of TD, such as coprophenomena, self‐injurious behaviors, and blocking tics, have been similarly re‐evaluated and are now interpreted as indicators of FTLB, especially if the cited diagnostic checklists are implemented rigidly.^10^, ^14^, ^22^, ^35^, ^36^, ^37^

It is therefore important to recognize that more research is needed to justify this shift in perspective. It is entirely possible that this new approach to TD tic phenomenology as exclusively characterized by predominantly simple tics with a slow onset demonstrates greater clinical validity than the previous inclusive definition—that it, for example, promotes a more accurate conceptualization of neurodevelopmental tics, is more in line with the clinical experience, or is more useful for both clinicians and patients. However, considering the impact that this change could have on the evaluation and use of previous research, the treatment of patients, and the general conceptualization of TD, it seems imperative that it is not implemented too quickly and before further discussion has been completed.

#### Validity of Demographic Variables

In the FTLB literature, the patients’ demographic details are ascribed a high level of diagnostic value. In many articles, older age at symptom onset, female sex, presence of specific psychiatric comorbidities, psychosocial problems at home, and presence of other FNDs are pointed out as key factors setting FTLB patients apart from patients with TD.^1^, ^6^, ^13^ As such, these details are also included in the diagnostic checklists, both of which include a cut off of 12 for age at onset and the presence of anxiety and depression as comorbidities, while Trau and colleagues also include female sex.^10^, ^14^

However, the validity of these demographic factors may have the same problem of overlap between the patient groups as the phenomenology. It has been consistently shown that there is a subgroup of patients with TD who are female, have depression and anxiety as comorbidities, and develop tics after the age of 12, just as there exist patients with FTLB to whom none of this applies.^3^, ^7^, ^37^, ^38^ Furthermore, there might be a risk that these general differences in demography have been created by clinicians’ bias towards categorizing the symptoms of young, female patients with depression and anxiety as functional and/or result from the aforementioned circular reasoning. While there is currently no clear evidence of such a bias in the FTLB literature, it is well‐documented in the wider FND literature, which shows that being a young woman with a mood disorder confers the greatest risk of FND misdiagnosis. The wider FND literature therefore encourages reliance on only clinical symptoms in the diagnostic process.^39^, ^40^, ^41^, ^42^ Considering the weight these demographic factors are currently given, it is important that their inclusion in the diagnostic checklists and patient characteristics is based on clinical research data and that their value as an indicator rather than a strict criterion is highlighted.

### Implications for Classification Systems

Overall, a diagnosis can be driven by two premises: its clinical usefulness and its consistency with the available research data. The previous sections of this article have focused on the possible influence of circular reasoning and bias on the conceptualization of FTLB and the potential problems with overlap between FTLB and TD patient characteristics, which may negatively impact clinical usefulness. In terms of its consistency with the available research, the diagnosis of FTLB might also benefit from further refinement in light of existing classification systems.

Although there has been extensive work focusing on delineating the traits of FTLB as its own entity, there has yet to be any attempt at placing FTLB within the context of the wider TD or FND literature or to use this wider literature to inspire research or inform the understanding of FTLB. In the context of the current FTLB definition, it is difficult to categorize it as a FND based on the wider FND literature. In both the DSM‐5 and ICD‐11, a mandatory criterion for a diagnosis of a FND is the presence of a positive sign.^24^, ^25^, ^43^, ^44^ A positive sign is a symptom that is incongruent with the accepted biological understanding of the disorder, for example due to internal inconsistency or due to the presence of characteristics that should be impossible. Examples are Hoover's leg sign which is used in the diagnosis of functional leg paralysis, or the clean EEG seen during episodes of psychogenic non‐epileptic seizures (PNES) in contrast to the epileptiform abnormalities seen during organic seizures.^45^ While it is well‐established in pediatrics that stress can trigger or worsen symptoms without necessarily eliciting a positive sign (eg, in primary headache), the presence of a positive sign that indicates incompatibility with a recognized neurological disease is mandatory for a diagnosis of an FND.

In the FTLB literature, it has proven challenging to identify reliable positive signs, which means that FTLB is not fully aligned with the diagnosis of an FND as defined in the DSM‐5 and the ICD‐10.^24^, ^25^, ^43^, ^44^ Previous practices of establishing an FND diagnosis based on atypical symptom presentation or patient demography have been largely abandoned, leaving presence of the positive sign as the only valid and relatively objective diagnostic criterion.^39^, ^40^, ^41^, ^42^, ^45^ In itself, the lack of a positive sign confirming the diagnosis of FTLB is not a problem, as the diagnosis could still serve as a perfectly viable clinical tool despite these limitations. However, as previously mentioned, clinical usefulness of the diagnosis of FTLB is also limited. As such it seems important to explore and characterize FTLB's place within the larger FND literature.

FTLB's lack of a positive sign and the difficulties with placing it within the FND literature may largely be attributed to the characteristics of TD. Tics as the central symptom of TD not only have many different expressions but also closely resemble voluntary movements and vocalizations which may lead to difficulties distinguishing tics from non‐tic behaviors. Furthermore, the attributes of TD also include many of the traits traditionally used as positive signs such as internal inconsistency. While internal inconsistency could be used to set FTLB apart from TD, to so do would be challenging as TDs are also naturally inconsistent with many patients being distractable or triggered by specific situations.^30^, ^46^ The lack of a complete neurobiological understanding of TD further complicates this issue, as it makes it difficult to ascertain which symptoms the underlying mechanism could realistically produce.^47^ This incomplete understanding means that it is currently impossible to find a positive sign or biomarker for TD removing the possibility of a gold standard test for the differential diagnosis between TD and FTLB. Thus, to clarify the issues surrounding FTLB and particularly its place in the FND literature, further exploration of the neurobiological mechanism behind TD and particularly which phenomenology it would be impossible for this pathological mechanism to produce would be beneficial.

### Future Perspectives

In the beginning of the COVID‐19 pandemic, clinicians all over the world realized that a new patient group had appeared within the realm of tic disorders. Researchers and clinicians sought to characterize this new group, a clinical exercise that eventually led to the development of checklists for the diagnosis of FTLB. As described in this article, the diagnosis of FTLB might be challenged by circular reasoning, lack of a positive diagnostic sign, overlap between the described demography and phenomenology of FTLB and TD, and co‐occurrence of both phenomena in a single patient. The overlap in patient characteristics between the groups indicates that more work is needed to identify the characteristics which clinicians might effectively use to categorize patients as having FTLB. As the diagnostic process of experienced clinicians is often more a case of implicit heuristic pattern recognition than an explicit evaluation against set criteria,^48^ pinpointing which characteristics are used in this process is often difficult. Clarification of these characteristics is particularly important if the diagnosis is to be used by clinicians with less experience.

One future direction in this regard could be to take a closer look at the specific characteristics and context of the FTLB groups’ symptoms. While, for example, patients with both FTLB and TD may exhibit coprophenomena and other complex movements and vocalizations, these symptoms may differ qualitatively, in their absolute number, or in terms of when and where they appear. If so, in depth descriptions of these differences may be helpful.

Another way forward could be to review which important characteristics have been found in other FND patient groups, as patients with FND do tend to share characteristics across different manifestations of the disorder and may even switch from one FND to another.^15^, ^44^ Furthermore, as circular reasoning during diagnosis formulation is a common problem within the FND field, other researchers have developed complementary ways to create diagnostic criteria, for example through examining a wide range of different symptoms and using cluster analysis to identify specific syndromes, a method which could be potentially relevant for FTLB.^49^

A different approach to identify potential clinical characteristics of FTLB would be to increase research into the underlying mechanism of TD and its relation to tic phenomenology in order to determine which behaviors this mechanism would be unable to produce and therefore could be used as characteristics of FTLB. Overall, there may be a need for further discussion among experts to achieve a more fine‐grained characterization of the symptoms of FTLB.

## Conclusion

In conclusion, more research and discussion are needed to fully and reliably differentiate the diagnoses of FTLB and TD. This research may include detailed qualitative characterizations of the behaviors displayed by each patient group, explorations of overlaps between FTLB and other FND patient groups, or cluster analyses of a wide range of characteristics in a FTLB and TD population to determine which traits set them apart. A different option would be to explore the mechanism behind TDs to enable localization of positive sign, as better understanding of this mechanism would improve our ability to determine which symptoms it would not be able to create. Furthermore, it is important to highlight the nuance of symptom overlap between TD and FTLB patients to new, less experienced clinicians.

## Author Roles

(1) Article development: A. Conception, B: Initial development; (2) Manuscript: A. Writing of the first draft, B: Review and critique.

K.A.: 1A, 1B, 2A

A.C.: 2B

N.Z.: 2B

K.M.W.: 2B

T.H.: 2B

L.S.: 1B, 2B

N.D.: 1B,2B

## Disclosures

**Ethical Compliance Statement:** Informed patient consent or formal ethical approval was not necessary for this work. We confirm that we have read the Journal's position on issues involved in ethical publication and affirm that this work is consistent with those guidelines.

**Funding Sources and Conflicts of Interest:** No specific funding was received for this work. The authors declare that there are no conflicts of interest relevant to this work.

**Financial Disclosures for the Previous 12 Months:** In the last 12 months KA has received project funding from Rigshospitalets Fond and from the Danish Childhood Cancer Foundation. In the last 12 months KM‐V has received financial or material research support from DFG: GZ MU 1527/3–1 and GZ MU 1527/3–2 and Almirall Hermal GmbH. She has received consultant's and other honoraria from Canymed, Emalex, Eurox Group, Sanity Group, Stadapharm GmbH, Swiss alpinapharm, Synendos Therapeutics AG, Tetrapharm, and Triaspharm. She is an advisory/scientific board member for Branchenverband Cannabiswirtschaft e.V. (BvCW), Sanity Group, Synendos Therapeutics AG, Syqe Medical Ltd., and Therapix Biosciences Ltd. She has received speaker's fees from Almirall, Bundesverband der pharmazeutischen Cannabinoidunternehmen (BPC), Cogitando GmbH, Emalex, Grow, Medizinischer Dienst Westfalen Lippe, Noema, streamedup! GmbH, and Vidal. She has received royalties from Elsevier, Medizinisch Wissenschaftliche Verlagsgesellschaft Berlin, and Kohlhammer. She is an associate editor for “Cannabis and Cannabinoid Research” and an Editorial Board Member of “Medical Cannabis and Cannabinoids” und “MDPI‐Reports” and a Scientific board member for “Zeitschrift für Allgemeinmedizin.” NS has participated in clinical trials supported by Emalex. She received scientific grants from the Polish Ministry of Health, Tourette Association of America, American Academy of Neurology and American Brain Foundation. She received speaker honoraria from Biogen. NMD has received speaker honoraria from Roche Pharmaceuticals A/S.

## Data Availability

Data sharing not applicable to this article as no datasets were generated or analysed during the current study.
